# Research on Spatial-temporal Spread and Risk Profile of the COVID-19 Epidemic Based on Mobile Phone Trajectory Data

**DOI:** 10.3389/fdata.2022.705698

**Published:** 2022-04-27

**Authors:** Qi Zuo, Jiaman Du, Baofeng Di, Junrong Zhou, Lixia Zhang, Hongxia Liu, Xiaoyu Hou

**Affiliations:** ^1^Institute for Disaster Management and Reconstruction, Sichuan University, Chengdu, China; ^2^The School of International Studies, Sichuan University, Chengdu, China; ^3^Chengdu Fangwei Technology Co., Ltd., Chengdu, China; ^4^Sichuan Wisesoft System Integration Co., Ltd., Chengdu, China; ^5^West China Hospital, Sichuan University, Chengdu, China; ^6^SinoMaps Press Co., Ltd., Beijing, China

**Keywords:** COVID-19, community, risk profiling, mobile phone trajectory data, the spatiotemporal spread

## Abstract

The COVID-19 epidemic poses a significant challenge to the operation of society and the resumption of work and production. How to quickly track the resident location and activity trajectory of the population, and identify the spread risk of the COVID-19 in geospatial space has important theoretical and practical significance for controlling the spread of the virus on a large scale. In this study, we take the geographical community as the research object, and use the mobile phone trajectory data to construct the spatiotemporal profile of the potential high-risk population. First, by using the spatiotemporal data collision method, identify, and recover the trajectories of the people who were in the same area with the confirmed patients during the same time. Then, based on the range of activities of both cohorts (the confirmed cases and the potentially infected groups), we analyze the risk level of the relevant places and evaluate the scale of potential spread. Finally, we calculate the probability of infection for different communities and construct the spatiotemporal profile for the transmission to help guide the distribution of preventive materials and human resources. The proposed method is verified using survey data of 10 confirmed cases and statistical data of 96 high-risk neighborhoods in Chengdu, China, between 15 January 2020 and 15 February 2020. The analysis finds that the method accurately simulates the spatiotemporal spread of the epidemic in Chengdu and measures the risk level in specific areas, which provides an objective basis for the government and relevant parties to plan and manage the prevention and control of the epidemic.

## Introduction

Since the first reported case of COVID-19 in the world in December 2019, and until 30 September 2020, 208 countries and regions on six continents have successively reported confirmed cases. The mortality rate of the European countries is peaking at more than 10% at the end of March 2020 (Niu et al., 2020). Studies have found that the spread of the COVID-19 virus is affected by population density and population mobility, and has obvious temporal and spatial aggregation (Huang and He, 2010; Ahmadi et al., 2020; Laborde et al., 2020; Li et al., 2020). Therefore, tracking and isolation of close contacts and suspected cases is the key to epidemic prevention and control.

Mobile phone trajectory data is a kind of spatiotemporal trajectory big data. Based on the position of the cellular base stations, the data can be extrapolated into the spatiotemporal distribution and flow matrix of the entire population in a highly anonymized manner. The rapid development and popularization of information technology make mobile phone trajectory data analysis become one of the most effective ways to quickly track the movement of the population. However, mobile phone trajectory data is complex and diverse, and the analysis workload is heavy. Fortunately, the rapid collision method based on time–space trajectory greatly improves the analysis efficiency. In the scene of large data volume and sizeable crowd, this method can still quickly analyze the data with high performance and obtain information about the number of encounters or contacts between individuals and the location of the contacts (Tang et al., 2017; Wang, 2017). Therefore, based on the characteristics of mobile phone trajectory data, by combining the communication signaling and geographic information, we simulate the full trajectory data of more than 16 million people in Chengdu to form the trajectory basis for spatiotemporal collisions. Process continuous trajectory data, find out potential high-risk populations and their distribution, classify and evaluate the activity area and risk size formed by the re-diffusion of potential high-risk populations, and analyze the spatial activity and spatial relationship of potential high-risk populations. Finally, through the risk assessment of these regions, the spatiotemporal spread and risk profiles of the epidemic are constructed, in order to provide a methodological basis for guiding the allocation of anti-epidemic resources and manpower in specific geographic regions and the formulation of epidemic prevention and control policies.

The potential risk population referred to in this article is the group of people who have had “time and space” intersections with the confirmed patients within 14 days.

## Literature Review

Studies have found that the spread of the COVID-19 virus, affected by population density and population mobility, displays obvious Space-time aggregation (Ahmadi et al., 2020; Laborde et al., 2020; Li et al., 2020; Huang and He, 2010). At present, the research on the prevention and control measures of COVID-19 mainly focuses on: rapidly tracing the movement of contacts within 4 days of the onset of the COVID-19 symptoms is critical for epidemic prevention and control (Willem et al., 2020), but the ultimate success of epidemic prevention and control depends on the speed and effectiveness of controlling suspected infected persons. Only when the time from primary case infection to contact tracing is shorter than the incubation period can the spread of the outbreak be prevented to the greatest extent possible (Hsiang et al., 2020; Keeling et al., 2020). Besides appropriate social isolation can reduce the COVID-19 infection rate, mortality rate, and the need for medical resources (Aaa, 2021). For example, in socially intensive environments such as schools or workplaces (Ahmed et al., 2018). However, with the advancement of current vaccine research and development technology, contact tracing and follow-up control measures have become particularly important in the early stages of epidemic control (Müller et al., 2000; Kwok et al., 2019; Read et al., 2020; Walker et al., 2020). Rapid and effective contact tracing can reduce the initial number of cases, which would make the outbreak easier to control overall (Jin et al., 2020). Effective contact tracing and isolation could contribute to reducing the overall size of an outbreak or bringing it under control over a longer period (Hellewell et al., 2020; Xu et al., 2020).

They used mobile-phone-data to develop a spatio-temporal “risk source” model that leverages population flow data to forecast confirmed cases, and identify high-transmission-risk locales at an early stage (Jia et al., 2020). Or they used the location data of the subscriber identity module (SIM) card from the largest mobile phone company in Haiti, to predict the scale and trend of population migration in Haiti following the 2010 earthquake and cholera outbreak. His studies have shown that it is potentially efficient to use this method to estimate population mobility in areas with high mobile phone usage (Bengtsson et al., 2011). Since the outbreak of the COVID-19 epidemic, many scholars have also done relevant theoretical and applied research on the protection of the epidemic and the resumption of work and production based on mobile phone signaling and spatiotemporal data. For example, based on epidemiological survey data of COVID-19 infection cases and mobile phone trajectory data of 15 million users during the epidemic, the spatiotemporal characteristics of COVID-19 and changes in personnel mobility were analyzed (Li et al., 2020; Liu et al., 2021). By aggregating and analyzing the big data and the epidemic data, it suggests cutting off the connection between different human mobility network communities and blocking the local transmission inside the community (Dong et al., 2020). The Johns Hopkins University in the United States has developed a real-time updated global spatiotemporal database of COVID-19 for epidemic prevention and research. The Chongqing has also formed a COVID-19 epidemic query system based on natural resources, a spatiotemporal geographic database, and a spatiotemporal big data service platform, and developed a COVID-19 epidemics map to report the progress of the epidemic in real-time. In addition, the research also builds a population mobile network model based on mobile phone location data to assess the possibility of infection in different regions. And with the support of spatiotemporal data, a comprehensive analysis of the spatiotemporal distribution of patient behavior trajectories was carried out. In addition, combining official data onto city, district, and county governments. Based on official case data, explore the temporal and spatial evolution of the COVID-19 epidemic and assess the epidemic risk level of key cities (Liu et al., 2020; Wang et al., 2020).

Previous research results have effectively supported COVID-19 epidemic protection and control of the epidemic at the national, social, and urban levels, but it needs to be in-depth, which has played an important role in blocking the spread of the epidemic, ensuring the needs of residents, and maintaining social stability community's research. In addition, most of the discussions on COVID-19 epidemic protection and control measures focus on the isolation and control of two types of people, confirmed patients and contacts, and how to quickly identify and locate the activity trajectories of these two groups of people, and take accurate and timely follow-up prevention and control measures. There are some shortcomings, and these two points are the main content that this article wants to explore.

## Methods

### The Research Data

The data used in this article come from the epidemic community data released by the Sichuan Provincial Health and Health Commission from 15 January to 15 February 2020, and questionnaire data of 10 confirmed cases in a hospital in Chengdu. Interviews include age, gender, residence, working place, time of diagnosis, time of isolation, travel, transportation, and relationship with other existing confirmed cases, etc. It also includes statistics on the population of more than 16 million in Chengdu.

### The Research Methods

#### Method to Generate the Population Activities

1) Signaling location data integration: According to the time and space distribution characteristics of the signaling trajectory data of a single user entity, integrate the adjacent signaling data in time and space to correct the error data and eliminate invalid data. Determine the characteristics of the signaling trace data of the user in a behavior scenario;
2) Space block entity feature trajectory calculation: According to the base station cell engineering parameters, including network standard, coverage type, coverage scene, direction angle, latitude and longitude coordinate position, etc., combined with the base station coverage gravity model and cluster analysis in the actual scene, the trajectory data is processed. Calculate the user's actual geographic location; use the actual location and coverage coordinate distribution of the space block provided by China Map Publishing Group to calculate the user's characteristic trajectory on the space block entity;
3) User space behavior recognition: According to the user's behavior characteristic trajectory on the spatial entity, combined with the attribute characteristics of the context space block entity in the trajectory, a user behavior trajectory chain is formed;
4) User base location tags and behavior feature tags: According to the user behavior tag trajectory chain, the multi-dimensional statistical results of user behavior characteristics are formed, such as users, blocks, behavior frequency within a period, average residence time, number of days of stay in working days, non-work analysis indicators such as the number of days of stay in a day, the number of similar behavior blocks; At the same time, a variety of model algorithms including Monte Carlo tree search are introduced to label users with basic location—workplace, residence, etc.; combined with analysis indicators to characterize Various feature tags form a clear user portrait.

#### Model Structure

We suppose the operator's population trajectory data: *P*_*i*_, $\overset{t}{\overset{︷}{\left\{l_{x}\ldots l_{i}\right\}}}$, where “*P*_*i*_” is a person, “*l*_*i*_” is a location, “$\overset{t}{\overset{}{\overset{︷}{}}}$” is time and time span. Finally, the spatial-temporal collision forms the population trajectory data.

**Step 1: Conduct basic evaluation of location-based on population spatial activity behavior**.

1) Based on the trajectory of human activities, the stationary points of people is deduced, where “*p*_*i*_” is a person, “*p*_*i*_,$\overset{t}{\overset{︷}{\left\{l_{x}\ldots l_{i}\right\}}}$” is the trajectory of “*p*_*i*_”. The effective stationary position of “*p*_*i*_” is “*p*_*i*_{*L*_1_…*L*_*i*_}”, where “*L*_*i*_” represents the effective lingering position obtained based on the statistical cycle of “$\overset{t}{\overset{︷}{\left\{l_{x}\ldots l_{i}\right\}}}$”, and the lingering time is more than 30 min.
2) According to “*p*_*i*_*sb*, {*L*_*live*_…*L*_*i*_ }”, “p_i_” is deduced, where “*a*_*i*_” is the activity of “p_i_” and “d_i_” is the dispersion of lingering position of “p_i_” (“a_i_” is the locations that people have visited and lingered in the target cycle; “*d*_*i*_” is the calculation of the degree of location dispersion involved in “*p*_*i*_*,{L*_*live*_…*L*_*i*_*}*”, i.e., the degree of lingering points dispersion of “*p*_*i*_”).
3) Table of spatial behavior data of a new person —TAB1;
4) Taking “*L*_*i*_” as the statistical objective, “*L*_*i*_, *P, P*_*live*_, *A, D, F*—*TAB2*” is derived, where “*P*” is the population size: the number of people effectively linger at “*P*_*all*_” is the de-duplication count of “*p*_*i*_”, and “*P*_*live*_” is the resident population: when the number of people effectively resident in “*L*_*live*_” is the de-duplication count “*p*_*i*_” etc.; “*A*” is population activity: $\bar{a}=\frac{1}{n}\overset{n}{\underset{i=1}{\sum }}a_{i}$, “*D”* is the diffusance: what other locations have been visited by people who has stayed at this location, and then set this location as the center to calculate the dispersion of the two locations, “*F*” is fluidity: “$\frac{P_{live}}{P_{all}}\;*\;100\%$”.

**Step 2: Assuming that the specific patient is known, the risk factor is determined at the increased position**.

Define “*S*” as the identified risk, “*s*_*i*_” as the specific patient, and “*TAB1*$=p_{i}^{{}^{\prime }}=p_{i}^{{}^{\prime }}$, $\overset{t}{\overset{︷}{\left\{l_{x}\ldots l_{i}\right\}}}$”, based on “*s*_*i*_”.

1) In TAB1, based on “*S(*$p_{i}^{{}^{\prime }}$*)*”, get “$p_{i}^{{}^{\prime }},\left\{L_{live}\ldots L_{i}\;\right\},a_{i}^{{}^{\prime }}{,d}_{i}^{{}^{\prime }}$—TAB3.”
2) “{*L*_*live*_…*L*_*i*_}” in TAB3, use “*L*_*i*_” as the statistical target to calculate “$L_{i},P^{{}^{\prime }},{P_{live}^{{}^{\prime }},A}^{{}^{\prime }},D^{{}^{\prime }}$–TAB4”, where “*P*′” is the total number of patients living in this location, “*A*′” is the patient's spatial activity, and “*D*′” is the patient's spatial spread.

**Step 3: By the rapid collision method based on the trajectory of patients passing by at the same time, increase the potential risk factors at the location**.

1) Based on “*t*” and “*l*_*i*_” in “$p_{i}^{{}^{\prime }},\overset{t}{\overset{︷}{\left\{l_{x}\ldots l_{i}\right\}}}$” of “*s*_*i*_”, map “*p*_*i*_,$\overset{t}{\overset{︷}{\left\{l_{x}\ldots l_{i}\right\}}}$” to get “$p_{i}^{{}^{\prime \prime }}$” (excluding “$p_{i}^{{}^{\prime }}$”).
2) In TAB1, get “$p_{i}^{\prime \prime },\left\{L_{live}\ldots L_{i}\right\},a_{i}^{{}^{\prime \prime }}{,d}_{i}^{\prime \prime }$, —TAB5” based on “*B(*$p_{i}^{{}^{\prime \prime }}$*)*”
3) In TAB5, “{*L*_*live*_…*L*_*i*_}” takes “*L*_*i*_” as the statistical target to get “$L_{i},P^{\prime \prime },A^{\prime \prime },D^{\prime \prime }$–TAB6”, where “{*L*_*live*_…*L*_*i*_}” (excluding “{*L*_*live*_…*L*_*i*_}” in *TAB*3), “*P*″” are the number of people who have been at the same time and space, “$P_{live}^{{}^{\prime \prime }}$” are the number of people who have been at the same time and space living here, “*A*″” is the spatial activity of people who have been at the same time and space, and “*D*″” is the spatial diffusion of people who have been the same time and space.

**Step4: Combine TAB2, TAB4, TAB6 to get TAB7, and comprehensively evaluate the risk category of the specific location based on TAB7**.

The location risk is preliminarily classified by using the numerical value, as shown in Table 1.

**Table 1:** Risk categories for a specific location.

**Category 1**	**Condition 1**	**Category 2**	**Condition 2**	**Instructions**
A Identified	P′> 0	Steady	P${}_{\text{live}}^{\prime }$> 0	The location of the confirmed cases can be identified by the people who live or linger here
risk		Fluctuation	P′> 0, P${}_{\text{live}}^{\prime }$= 0	
B Potential	P′= 0	Steady	P″ > 0, P${}_{\text{live}}^{\prime \prime }$ > 0	People who have been at the same time and space with the confirmed case, live here
risk		Fluctuation	P′= 0, P″ > 0, P${}_{\text{live}}^{\prime \prime }$ = 0	People who have been at the same time and space with the confirmed case, do not live here
C Normal	P′= 0, P″ = 0	Susceptible area	The A,D,F index is high	People in these areas move to many places and in a large range
areas		Other areas	The A,D,F index is low	People in these areas move to fewer places and in a small range

**Step 5: Based on Moran's I Statistics, conduct an overall evaluation of the risk distribution in the target area**.

Moran's I was used to measure the degree of Spatial autocorrelation (similar dimensional attributes), it means to evaluate whether the distribution of elements was aggregated, discrete or random. Moran's I index closer to “1” indicates aggregation, and closer to “-1” indicates dispersion. As shown in Table 2.

**Table 2:** Spatial autocorrelation instruction.

**Z(I)**	**Spatial distribution correlation**
>1.96	Positive spatial autocorrelation
[-1.96, 1.96]	Weak spatial correlation
< -1.96	Negative spatial autocorrelation

**Step 6: Based on “P**_**i**_**{L**_**1**_**…L**_**i**_**}”, get the specific spatial field correlation**.

Information entropy is used to measure the correlation of spatial fields. The conditions for defining the correlation index “r” between two discrete space fields are as follows: when the two space fields are independent of each other, there is “r_min_=0”. When the correlation degree of the two spatial fields increases gradually, “r” also increases gradually. There is “r_max_=1” when the two space fields are completely correlated. According to the nature of information entropy, the expression of “r” can be established.

**Figure 1:**
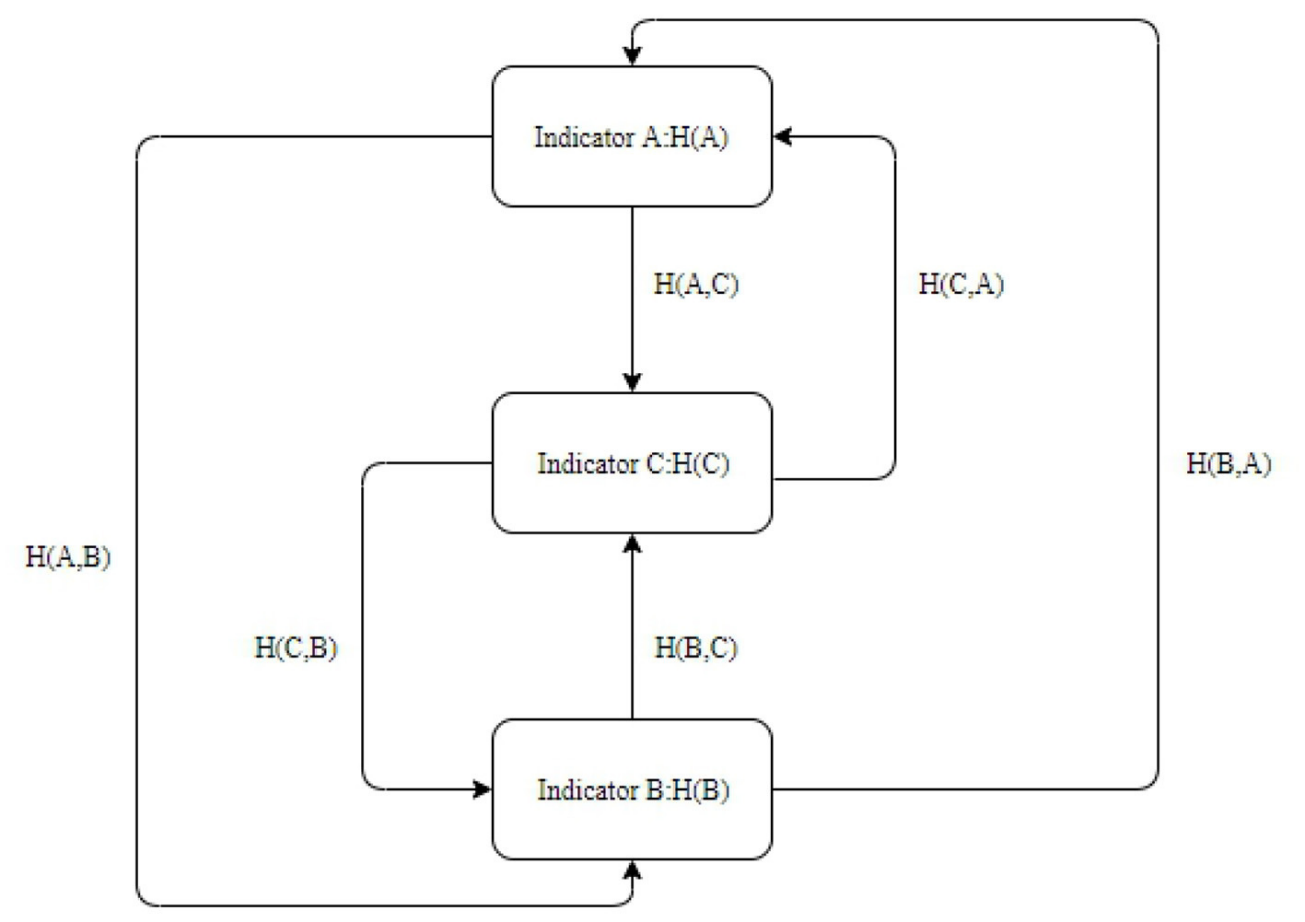
Information entropy relationship.

When the discrete state sets “A” and “B” are completely related, each type area of the two layers must have a one-to-one mapping relationship in space. According to the layer properties of the index “A” space, it is divided into “m” types. The proportion of the area of the type “ai” to the total area of the area is P(a_i_), and the layer properties of the index “B” space are divided into “n” types, and the proportion of the area of the type “b_j_” to the total area of the area is “P(b_j_).” “a_i_b_j_” represents the category after the type “a_i_” in the “A” layer and the space “b_j_” in the “B” layer are superimposed (up to “m^*^n” categories appear after the two layers are superimposed), and the area ratio is “P (a_i_b_j_),” and the information entropy is H(A), H(B) and H(A,B). Finally, the correlation index between thematic layers “A” and “B” is calculated. The higher the entropy is, the more information can be transmitted; the lower the entropy is, the less information can be transmitted. Thereby, the linkage cascading relationships among indicators of different dimensions can be classified. As shown in Figure 1.

## Results

### Conduct Basic Location Evaluation Based on Population Spatial Activity Behavior

#### Analysis of the Number of Population Resident Places

By using the trajectory activity data of 10 confirmed patients collected, the random distribution and activity spaces of more than 16 million Chengdu are simulated based on the model, and the number of residents places in Chengdu within 14 days is obtained. Whether it is a transient population or temporary population, the more effective residences there are, the higher the transmission of infection will be. As shown in Table 3.

**Table 3:** The number of effective population resident places.

**Classification (places)**	**1**	**2**	**3–4**	**4–5**	**6–10**	**>10**
Transient population (%)	17.30	9.13	5.61	3.12	2.66	0.01
Permanent population (%)	7.69	21.33	19.22	9.29	4.64	0.00
Total (%)	55.45		37.24		7.31	

*Transient population refers to the non-permanent population that appears during the monitoring period (14 days) and has effectively resided at least one place in the city*.

To classify the temporal and spatial activity of the population and set the risk coefficient. As shown in Table 4.

**Table 4:** Classify the temporal and spatial activity of the population and the risk factor.

**Spatial activity**	**Risk coefficient**	**Definition**	**Instruction**	
			**Normal people: Possibility of being infected**	**Patients: Transmissibility**
Low	1	Effective Resident Positions < =2 places	Low	Low
Medium	5	3 places < Effective Resident Positions <5 places	Medium	Medium
High	10	Effective Resident Positions >5 places	High	High

#### Analysis of the Scope of Population Activities

The analysis of the scope of activity is mainly aimed at the spatial distance of the “location,” that is, the description and analysis of the dispersion of the stationary points of people, which represents the direction of spatial diffusion. Combined with the number of resident positions, the spatial dispersion can be measured as whether it is concentrated or dispersed. The higher the value is, the higher its spatial propagation is. The setting of each propagation coefficient is shown in Table 5.

**Table 5:** The degree of spatial transmission of people.

**Number of position**	**Concentration (<1 km)**	**Dispersion (>1 km or negative spatial autocorrelation)**	**Random**
Number of position 1-2 (places)	1	2	Null
Number of position 3-5 (places)	3	4	4
Number of position >5 (places)	5	6	6

#### Distribution of Track Resident Point of People-Based on Resident Places

**Figure 2:**
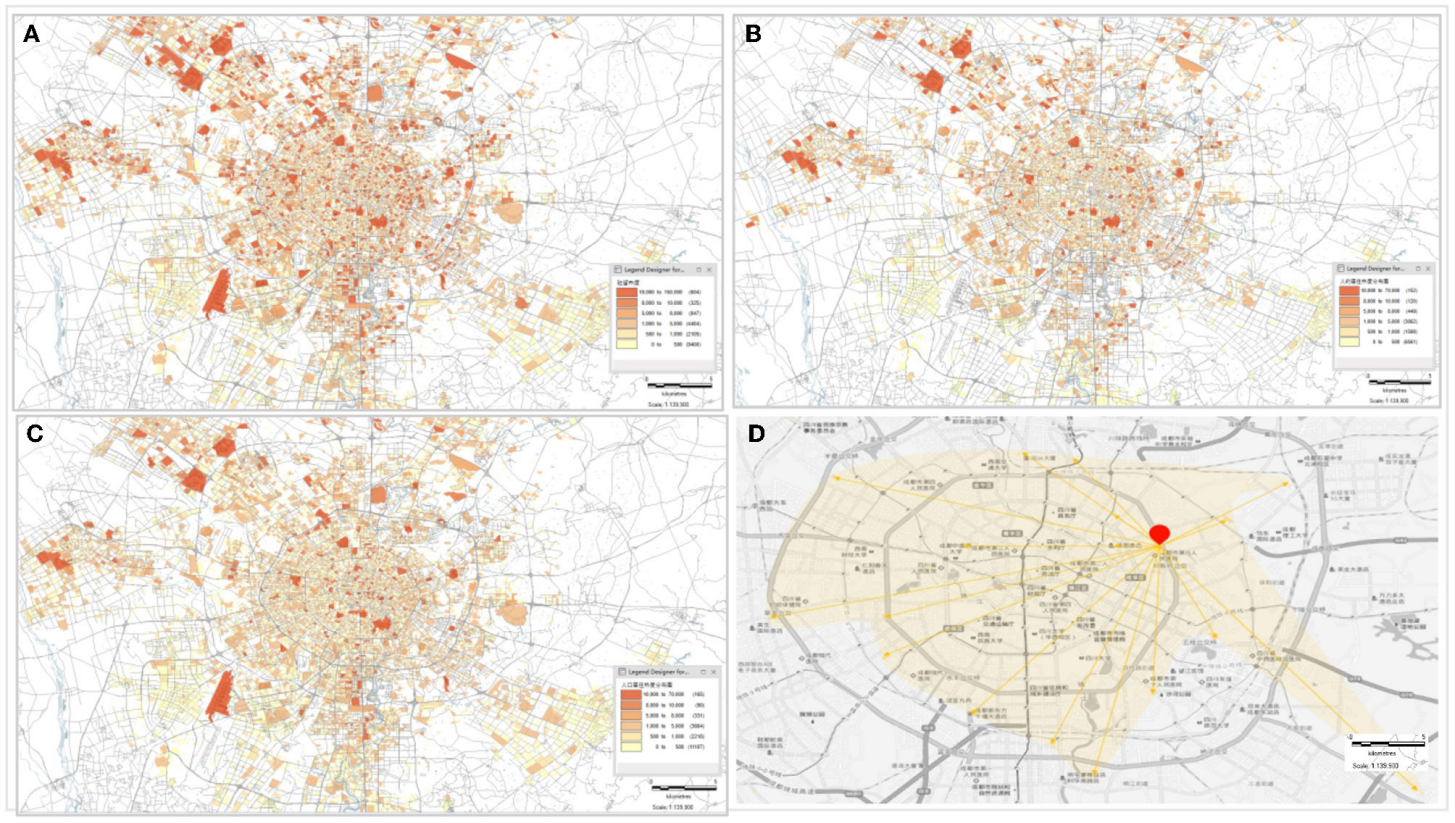
Distribution of track resident point of people **(A)** population resident heat distribution map **(B)** population residential heat distribution map **(C)** distribution of population spatial activity **(D)** distribution of population spatial. The darker color presents more people in the location, and the lighter color presents fewer people stay in the location, and correspondingly the heat is lower.

Based on the simulation data of population activity trajectory, the distribution of resident heat, residential heat, activity, and spatial diffusion of the population can be obtained. As shown in Figure 2.

It can be seen from the Population Resident Heat Distribution Map that the darker color presents more people stay in the place, and the lighter color presents less people stay in the place, and the resident heat are lower. Residential heat distribution is different from resident heat distribution, once a confirmed case is found in the place of residence, the size of the population at that location can be quickly known and the epidemic situation in the place can be protected in time. In addition, combining with “*P*,” location “*P*/*P*_*live*_” with high-population mobility can be quickly obtained. With high mobility (the greater the ratio), the higher the risk coefficient of transmission or infection in this location will be. In the Population Activity Distribution Map, the darker the color, the greater the number of related locations between this location and other locations. From the Distribution of Population Spatial Diffusion Map, the spatial diffusion of a single person is mainly spread out in the way of one point and more lines.

### Analysis of Spatial Activity of Confirmed Patients

#### Distribution of Residence and Track Resident Point of Confirmed Patients

**Figure 3:**
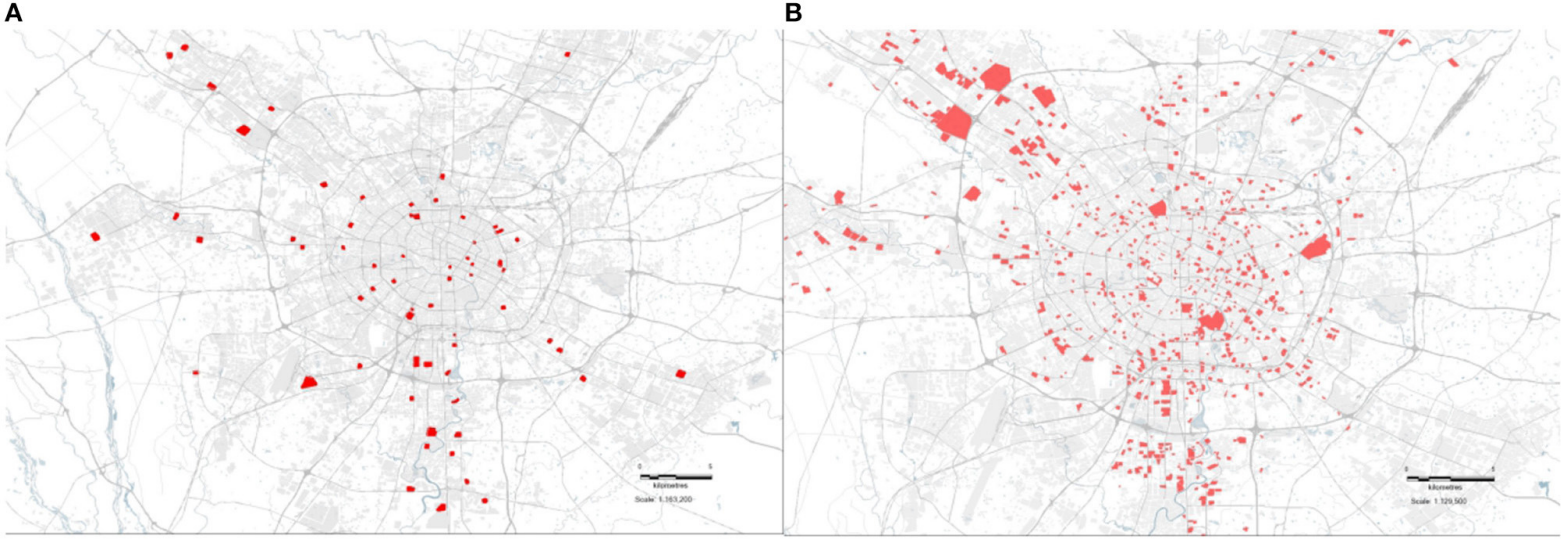
Distribution of residence and track resident point of confirmed patients **(A)** stationary place distribution **(B)** trajectory distribution.

According to the specific locations of the 94 closed communities with confirmed cases as of 14 February 2020 issued by the Health Commission of Sichuan Province, the specific location distribution map of the confirmed patients can be drawn, as shown in Figure 3.

As can be seen from the figure, the residents of the confirmed patients are mainly distributed downtown. Resident location distribution is related to regional population density and socio-economic vitality. The downtown or places with convenient transportation (subway, bus) lines, large shopping malls, and shopping centers are places where confirmed patients stay frequently.

#### Analysis of Activity and Spatial Diffusion of Confirmed Patients

The spatial information such as the patient's address and activity location is converted into spatial and temporal data to form the spatial transmission trajectory of the confirmed cases. According to the second step of the analysis method, the activity and spatial diffusion of the confirmed patients can be analyzed. However, in order to protect personal privacy, the trajectory of confirmed patients will not be analyzed graphically.

### Distribution of Track Resident Point of Trajectories of People, Who Have Been at the Same Time and Space With the Confirmed Patient

**Figure 4:**
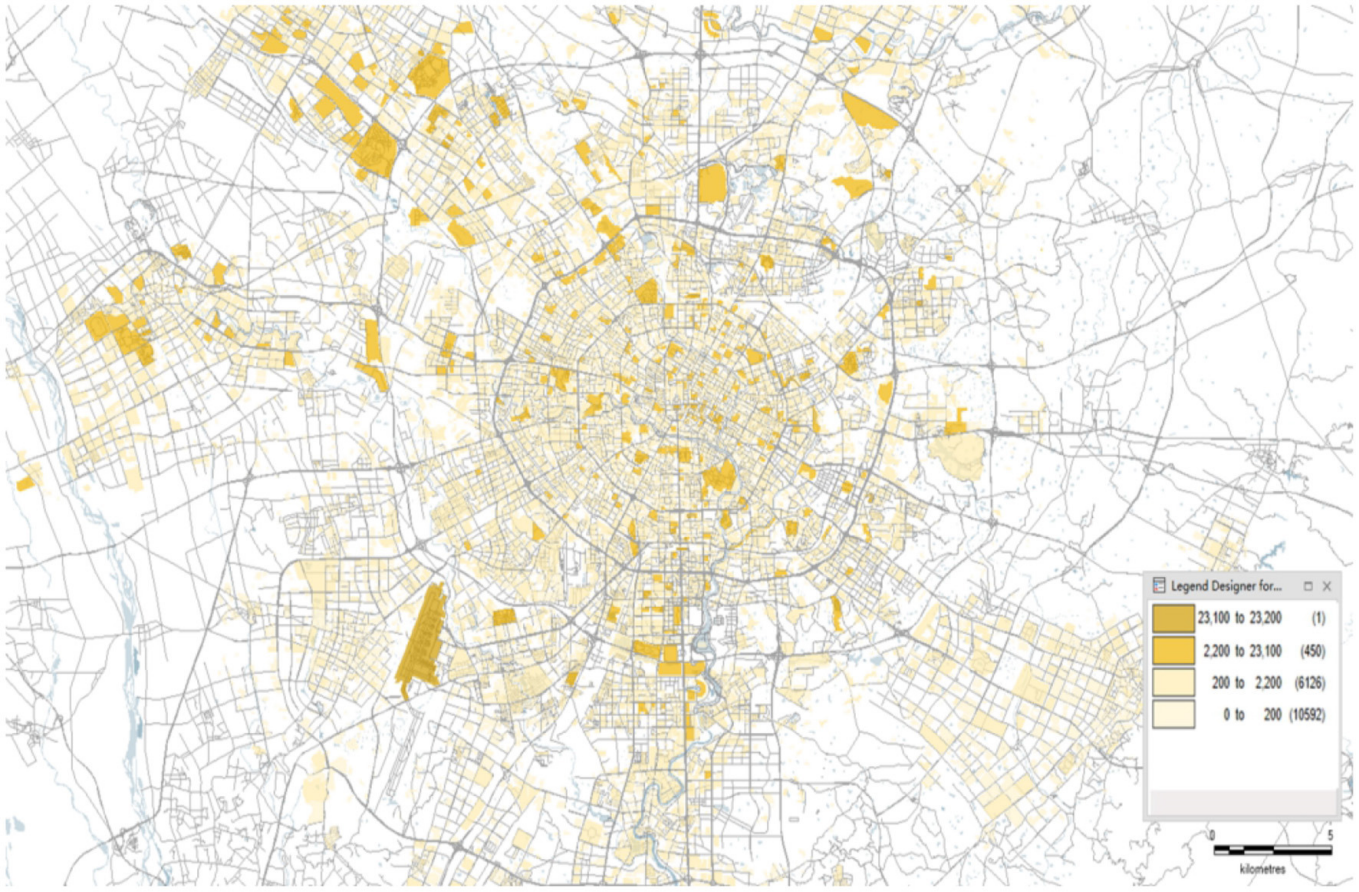
Distribution of track resident point.

According to Figure 4 the trajectories of people who have been at the same time and space with the confirmed patient, it can conclude that the trajectories of such people are almost all over the districts and counties of Chengdu. Among them, the densest track resident points are the downtown.

### Risk Profiling of COVID-19 Epidemic for Specific Location

As shown in Table 6.

**Table 6:** Calculation methods for different risk categories.

**Category 1**	**Color**	**Value at risk**
A Identified risk	Red	Number of people * Space Activity Coefficient * Propagation Coefficient
B Potential risk	Yellow	Number of people * Space Activity Coefficient * Propagation Coefficient
C Normal region	Green	Number of people * Space Activity Coefficient * Propagation Coefficient*Mobility

#### Calculation of Risk Factors for a Specific Location

Assuming that the risk value of each category is the same, the risk value of epidemic transmission is divided into segments, and the level is set. As shown in Table 5.

#### Analysis of Risk Categories at Specific Locations

**Figure 5:**
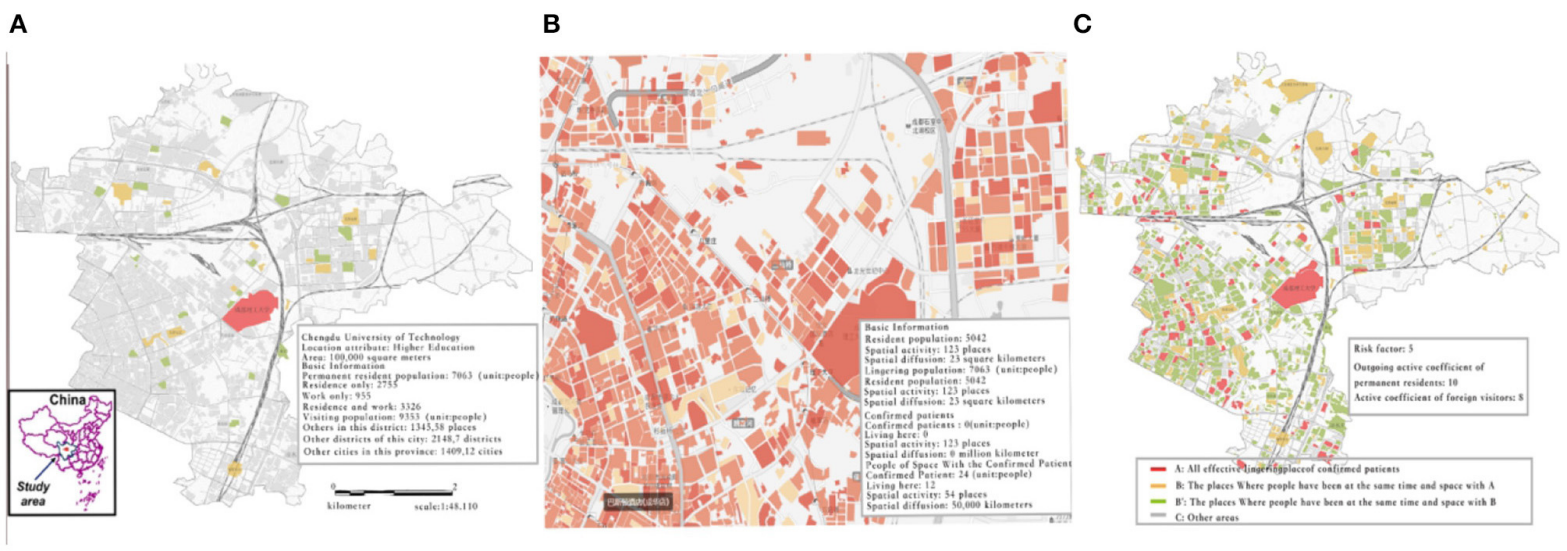
Comprehensive location evaluation of the Chengdu University of Technology **(A)** The Chengdu University of Technology geographical distribution. Based on the spatial activity of the population to realize the time and space traceability and time and space tracking: Be able to trace and track the source of time and space for residents (including residence only, work only, job, and residence at the same address/same street/same district), visits (non-resident population in the district); It can analyze the overall situation in the administrative area, each street, and the specific community. **(B)** Analysis of diagnosed patients and intermediate communicators of the Chengdu University of Technology **(C)** Several elements of risk assessment: Risk category: There are 3 categories (A, B, B). Risk factor: It is mainly obtained based on the comprehensive analysis of population number and regional population density. The larger the value, the higher the risk; Resident population's out-of-home activity coefficient: A factor of the risk coefficient, which describes the outdoor activities of the permanent population in the location. The larger the number, the more active the residents in the location are out-going activities; Inbound visitor activity coefficient: a factor of the risk coefficient, indicating the liquidity of the location, the higher the coefficient, the stronger the liquidity.

From 0:00 to 6:00 on 8 December 2020, 3 new cases of new coronary pneumonia were confirmed in Chengdu. The students of the Chengdu University of Technology have had contact with this confirmed case. Therefore, this article taking the Chengdu University of Technology as an example analyzes the comprehensive information of its location according to the aforementioned method as shown in Figure 5.

Combining calculations with specific models can be obtained, the Chengdu University of Technology, epidemic risk category: A, epidemic risk coefficient: 5, of which, the resident population's active coefficient of going out: 10, and the active coefficient of foreign visitors: 8.

## Risk Profiling of COVID-19 Epidemic in Chengdu

### Distribution of Regional Risks in Chengdu

According to the standardized statistics of the Local Moran's I index test, the spatial correlation values of different risk categories can be calculated, and the overall spatial distribution type can be determined based on this correlation, as shown in Table 7.

**Table 7:** Description of the spatial correlation distribution of different risk categories.

**Category 1**	**Correlation**	**Value specification**	**Result**
A Identified risk	1.12	Positive spatial	Concentration
		autocorrelation	
B Potential risk	1.28	Negative spatial	Dispersion
		autocorrelation	
C Normal region	−1.2	Weak spatial correlation	Random

According to the aforementioned formula, the spatial correlation distribution value of the identified risk area can be calculated. For areas with positive spatial autocorrelation, centralized protection deployment can be adopted. For areas with negative spatial autocorrelation, decentralized protection methods can be implemented. In areas with weak spatial correlation, the distribution among communities is generally random and the COVID-19 epidemic protection is complex. Therefore, the actual situation of the epidemic in the community should be investigated and corresponding protection measures can be taken.

### Portrait of Epidemic Risk in Chengdu

**Figure 6:**
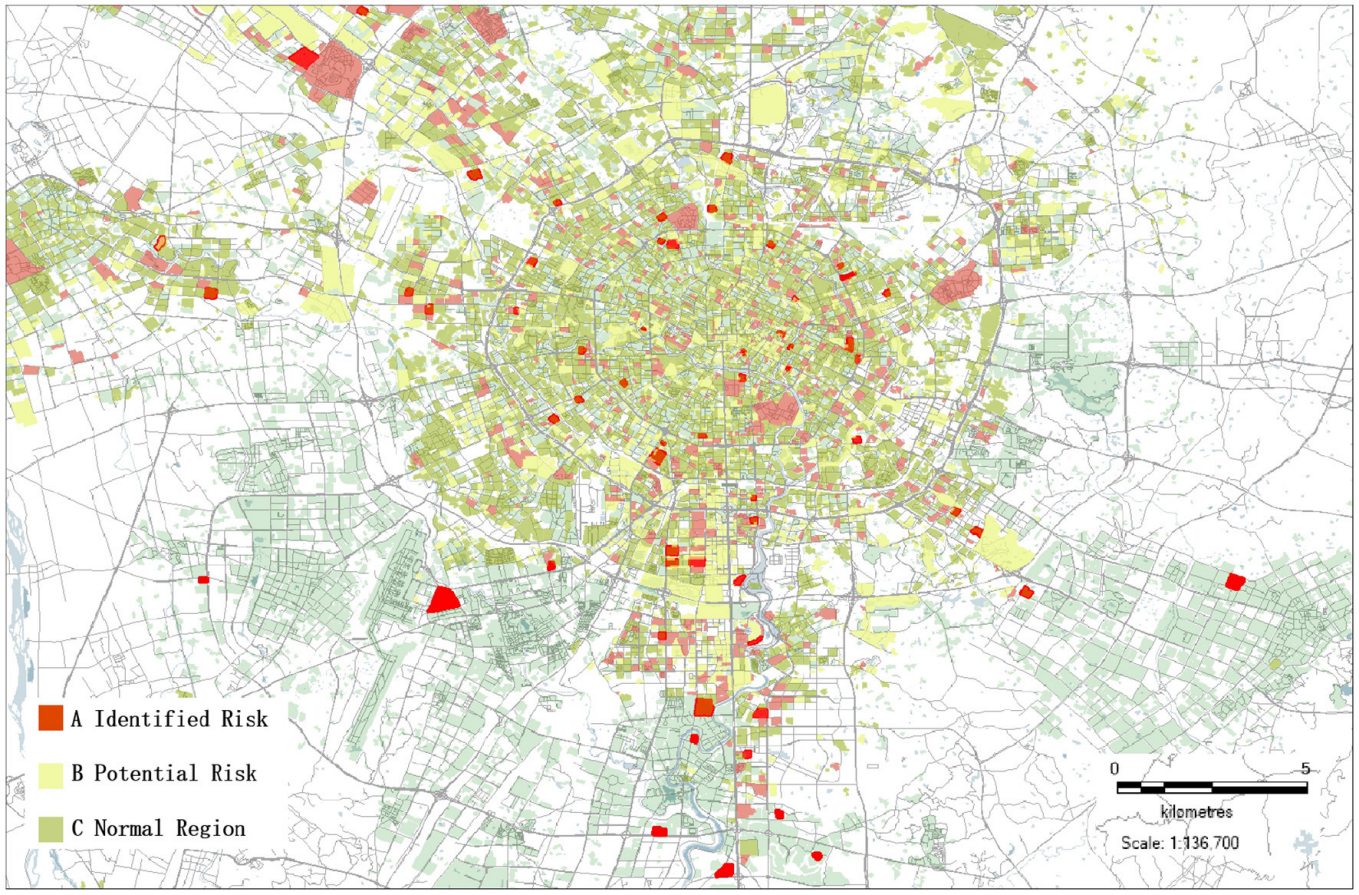
The COVID-19 epidemic risk map of Chengdu.

The risk value is segmented and the risk level is set. Assuming that the risk value of each category is the same, based on the comprehensive distribution, a geographic profile of the risk of the epidemic community in Chengdu can be obtained, as shown in Figure 6.

According to the different colors shown in the figure aforementioned, the risk of the COVID-19 epidemics in Chengdu can be clearly identified, and the sequence of nucleic acid testing for COVID-19 can also be accurately guided in each region. For example, in areas with high-spatial population activity, high diffusion, and high-transmission coefficient, priority will be given to arranging local professional epidemic prevention forces to the station in the community, and residents in the whole region should conduct nucleic acid testing. In the potential risk areas, nucleic acid testing and identification of suspected cases should be strengthened, and the movement of suspected cases should be closely tracked, which can help to cut off the transmission chain of the epidemic in the shortest time. In areas where the risk assessment shows normal, nucleic acid testing can be done voluntarily by residents, in order to promptly guide and relieve residents' nervousness.

### Analysis of COVID-19 Epidemic Spread in Chengdu

The analysis shows that 36% of the affected communities in Chengdu are within 1 km next to each other, and 9% of the communities are within 500 m. There are four main distribution characteristics: “A-B-C-A” triangular shape, “A-B-C” broken line, “A-B” segment type and scatter type, as shown in Table 8.

**Table 8:** Specific distribution characteristics of the affected communities.

**Distribution characteristics**	**Specific communities**	**Distance**
A-B-C-A triangular shape Distance <1,000 m	Zhonghai No. 9 Residence, Jinxi Hotel, Zhonghai Chengnan No. 1	445; 941; 835
A-B-C broken line Each section Distance <1,000 m	Lloyds Garden-Chaoyang Langxiang Plaza-Tianxin Garden	388; 921
	Hua Min Yiyuan-SiJi Kang Cheng Hotel-Shidai Haoting	604; 850
	Zhongliang Xiangyun-Wuhou Villa-Wuhou International Garden	521; 769
A-B, segment type Distance <1,000 m	Shuangnan Mansion-Shuangnan Garden Community	368
	Bali Yangguang-Xiangmulin Garden	468
	Jia Zhao Ye Li Jing Mansion-Ruijing Lanting	531
	Shenxianshu Courtyard Phase 4-Vienna International Hotel	536
	Fengjing Yaju-Gaoyi Hotel	596
	Tianfu Changcheng Bainanjun-Shidai Jincheng Hotel	849
	The Holy Birch City-Guandu East Road Alley	912
	Tianfu Oucheng-Qingjiang Huatng	922
	Wanke Golden Area-Junfa Shidai Junyuan	936
	Linjiang Road, Yard 8-Roland Hotel	974
	Jinsha Guoji-Shiji Jinsha	999

The distribution characteristics of the affected communities aforementioned in Chengdu: “triangle,” “broken line” and “line segment.” The causes and rules of the spread of the COVID-19 epidemic can be preliminarily summarized, so that different protection measures can be taken according to the distribution shape among communities.

### Spatial Distribution of Epidemic Communities in Chengdu

**Figure 7:**
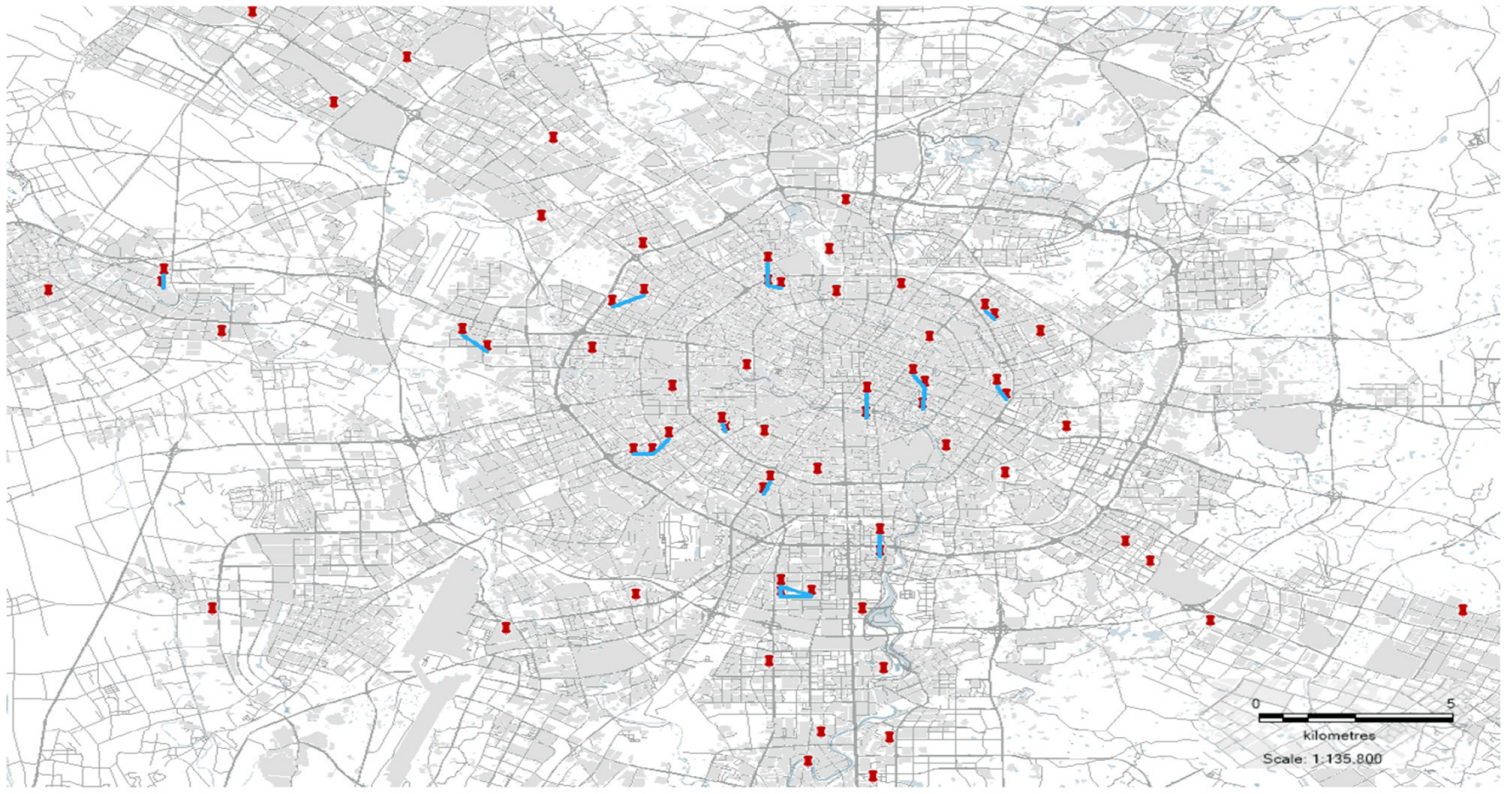
The spatial distribution of epidemic communities.

Based on the confirmed cases published by the Health Commission of Sichuan Province, the spatial distribution of the outbreak communities in Chengdu is described, as shown in Figure 7.

The aforementioned picture depicts the spatial distribution of the epidemic communities in Chengdu, which can guide relevant government departments to formulate appropriate plans to block the spread of the COVID-19 epidemics. In areas where epidemic communities are densely distributed, protection departments must implement strict epidemic surveillance to ensure early detection, quarantine, and treatment of infected people to reduce the spread of the COVID-19 epidemic. For non-infected communities that are far away from the affected communities, the protection strategy should be adjusted to prevent the importation of outbreaks from other places, so that the resumption of work and production can be carried out to the maximum extent.

## Discussion

Based on the mobile phone trajectory data, using the research methods and steps in this article, combined with the relevant data of the epidemic in Chengdu, the verification found that: according to the established model, based on basic population data and spatiotemporal collision methods, it accurately described people who have close contacts. The epidemic risk coefficient and risk level of the Chengdu University of Technology, and also the epidemic risk map of the entire area of Chengdu and the overall distribution characteristics of the epidemic community. This distribution feature can effectively guide different urban areas and risk communities to carry out nucleic acid inspections, putting anti-epidemic supplies, and formulating plans for resuming work and production.

In addition, this method can also be used for the research on spatial-temporal diffusion and profiling of various risks in the future. There are also aspects that need to be improved and optimized in the research: first, the activity trajectory of confirmed cases, medical information, and other data are relatively fragmented and not detailed enough. The overall profiling of the development of the COVID-19 epidemic in Chengdu may be biased. Future analysis of similar disaster events still needs to rely on more detailed spatial-temporal trajectory data and further study the micro-scale population activities and risk communication. Second, considering personal privacy and the difficulty of obtaining certain data, the analysis of the spatial diffusion of risk factors and the overall diffusion situation of the region is relatively rough. Therefore, the analysis method should be improved in the future, which requires a higher requirement for desensitization of spatial-temporal data.

## Data Availability Statement

The original contributions presented in the study are included in the article/supplementary material, further inquiries can be directed to the corresponding author/s.

## Ethics Statement

Ethical review and approval was not required for the study on human participants in accordance with the local legislation and institutional requirements. Written informed consent for participation was not required for this study in accordance with the national legislation and the institutional requirements.

## Author Contributions

QZ, BD, and JZ substantial contributions to the conception or design of the work and the acquisition and analysis. JZ, XH, HL, and LZ contributions to the acquisition and analysis of data for the work. JZ, LZ, and JD drafting the work or revising it critically for important intellectual content. QZ and BD final approval of the version to be published. LZ and JD agreement to be accountable for all aspects of the work in ensuring that questions related to the accuracy or integrity of any part of the work are appropriately investigated and resolved. All authors contributed to the article and approved the submitted version.

## Funding

National Key Research and Development Program of China, No. 2020YFD1100701.

## Conflict of Interest

JZ was employed by Chengdu Fangwei Technology Co., Ltd. LZ was employed by Sichuan Chuanda Zhisheng System Integration Co., Ltd. XH was employed by SinoMaps Press Co., Ltd. The remaining authors declare that the research was conducted in the absence of any commercial or financial relationships that could be construed as a potential conflict of interest.

## Publisher's Note

All claims expressed in this article are solely those of the authors and do not necessarily represent those of their affiliated organizations, or those of the publisher, the editors and the reviewers. Any product that may be evaluated in this article, or claim that may be made by its manufacturer, is not guaranteed or endorsed by the publisher.
